# Physician scatter dose in interventional CT fluoroscopy

**DOI:** 10.1002/acm2.14355

**Published:** 2024-04-14

**Authors:** Emily A. Knott, Jonathan L. Troville, Carlos Alberto Reynoso, Sean D. Rose, Martin G. Wagner, Fred T. Lee, Jeffery L. Radtke, Daniel R. Anderson, Annie Zlevor, Meghan G. Lubner, J. Louis Hinshaw, Timothy P. Szczykutowicz

**Affiliations:** ^1^ Cleveland Clinic Lerner College of Medicine Cleveland Ohio USA; ^2^ Department of Medical Physics University of Wisconsin‐Madison Madison Wisconsin USA; ^3^ University of Texas Health Science Center at Houston Houston Texas USA; ^4^ Department of Radiology University of Wisconsin‐Madison Madison Wisconsin USA; ^5^ Department of Urology University of Wisconsin‐Madison Madison Wisconsin USA

**Keywords:** fluoroscopy, interventional computed tomography, physician dose, radiation safety, scattered radiation

## Abstract

**Purpose:**

To quantify operator dose in interventional CT fluoroscopy (CTF) procedures and compare to catheterization laboratory and interventional radiology procedures.

**Methods:**

Fifteen studies quantifying operator scatter dose for c‐arm fluoroscopy and CTF were reviewed from a literature survey. The dose provided in each study was normalized to skin entrance air kerma and air kerma rate including backscatter. The average air kerma rate and exposure duration were calculated and compared between c‐arm fluoroscopy and CTF. To enable operator scatter dose calculations in CTF procedures, a model was created which scaled the dose from a reference condition.

**Results:**

Our literature survey indicated that for CTF relative to c‐arm fluoroscopy: (1) the mean air kerma rate is 3.5 times higher, while (2) the mean exposure duration is 31.6 times lower. On average, a physician would have to perform 9.1 times (i.e., 31.6/3.5) more CTF procedures relative to c‐arm fluoroscopy procedure to receive equal operator scatter doses. Our own experimental measurements provided scaling factors allowing physician scatter dose to be obtained at any rotation time, tube current, beam collimation, or beam energy.

**Conclusion:**

Our results shed light onto a currently mis‐understood issue in radiology. Namely, that physicians receive more dose from CT relative to c‐arm fluoroscopy motivating them to retreat from the room during interventional exposures. Our literature review and experimental measurements indicate the opposite it true; interventional CT produces on average nine times less physician dose relative to c‐arm procedures. Our method allows for prospective/retrospective determination of an individual's dose.

## 1 INTRODUCTION

Computed tomography (CT) is widely used as an image guidance modality for percutaneous interventions in the chest, abdomen, pelvis, and extremities. Since the first CT‐guided procedures were reported in 1976 by Haaga and Alfidi,^1^ substantial technical improvements have increased the breadth of procedures that can be performed, improved patient safety, and increased procedure speed. A particularly notable advancement was the development of slip ring helical CT which directly led to the introduction of CT fluoroscopy (CTF).^2^

CTF can be performed in both continuous mode (cCTF) and intermittent mode (iCTF). Concerns over high radiation doses with cCTF combined with the realization that real‐time imaging was rarely necessary led to the more recent introduction of intermittent CTF (iCTF), also known as “step‐and‐shoot,” “tap mode,” or “quick check” (e.g., SUREFLUORO for Canon Medical Systems USA, smartstep for GE Healthcare, i‐Fluoro for Siemens Healthineers). In iCTF, a single pedal tap results in a single tube rotation, a limited volume of images, and low radiation doses. However, confusion between the two CTF methods, inconsistent use of CT parameters (particularly beam collimation and mAs), and confusing radiation reporting nomenclature have led to a wide range of misconceptions related to CTF radiation exposure.^3^ Physician radiation dose is particularly complicated to capture without the use of highly sensitive real‐time radiation detectors placed at a consistent location and height beside the patient couch.^4^ This lack of clarity and consistency surrounding patient and physician radiation dose has likely slowed the adoption of “in‐room” CTF (i.e., cCTF or iCTF methods) despite known advantages in terms of procedure speed and patient outcomes. In many centers CT‐guided procedures are still performed with physicians walking in‐and‐out of the room after every needle reposition in a manner virtually indistinguishable from Haaga and Alfidi's initial description^1^ over 40 years ago.

The purpose of this study is to quantify physician scatter radiation dose during iCTF. The results are presented across a range of real‐world CT parameters and reported “per tap” so that physician exposure can be quickly estimated for individual procedures. We also perform a literature review to contextualize iCTF dose versus interventional procedures performed using c‐arm based fluoroscopy.

## 2 METHODS

### 2.1 CT scanner protocols and phantom

Measurements were taken using a multi detector CT scanner (Discovery CT750, GE Healthcare, Waukesha, WI) with a human sized phantom placed at isocenter (Mercury Phantom 4.0, Sun Nuclear Corporation, Middleton, WI). The 31 cm section of the phantom was scanned at 80, 100, 120, and 140 kV using 1.25, 2.5, 5, 10, 20, and 40 mm wide beam collimations with and without a 0.5 mm lead apron placed over the dose meter. All scans were acquired with a gantry rotation time of 1 s and the mA was adjusted for each kV to the highest possible value allowed by the scanner. All scatter dose measurements were subsequently normalized to 100 mAs. Scans were repeated six times to calculate mean and standard deviation. Error bars on all plots are standard error unless otherwise noted.

### 2.2 Dose measurement and analysis

**FIGURE A1:**
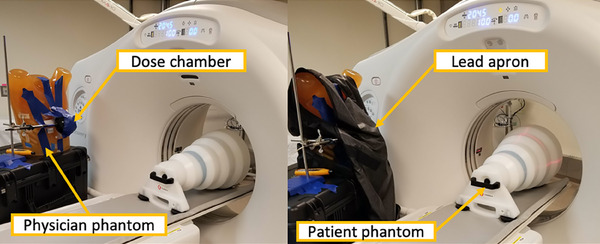
Experimental set up showing the dose chamber unshielded (left) and shielded (right) using a lead apron.

Dose was quantified using entrance air kerma (EAK), measured with a 12 cm spherical ionization chamber (800 cc collection volume; Extradin A6, Standard Imaging, Middleton, WI) and an electrometer (MAX‐4000, Standard Imaging, Middleton, WI). The chamber was placed to the side of the CT table to mimic the typical position of a physician's torso as shown in Figure A1. A phantom (“LUNGMAN” Kyoto Kagaku Co., Ltd., Fushimi‐ku Kyoto, Japan) was placed behind the chamber to provide backscatter, mimicking a clinical set‐up. The chamber was 125 cm from the floor, 54 cm from the center of the CT couch (measured orthogonal from the long axis of the couch), and 85 cm from the scanner iso‐center as shown in Figure A1.

### 2.3 Determination of operator scatter dose

Appendix A describes a method for experimental parametrization of operator scatter dose measurements allowing for the calculation of a physician's dose at any beam tube current, rotation time, beam collimation, and beam energy. An easy to follow “worksheet” is provided in Appendix A to facilitate readers performing their own dose calculation. Briefly, our method scales the dose from a reference condition with a dose of $D_{ref}$ and sums doses for each iCTF tap. Scaling accounts for changes in mA, rotation time, collimation, beam energy, short versus full scan geometry (see Appendix A for a description of short versus full scanning), and the presence of a lead apron. The reference condition dose was measured using no lead apron at 120 kV with a collimation of 10 mm, a full scan geometry, 100 mA and a rotation time of 1 s.

### 2.4 Calculation of physician scatter dose

The methods defined in Appendix A were used to calculate the physician scatter dose from a typical interventional procedure performed using iCTF at UW‐Madison. To define a typical procedure at UW‐Madison, we pulled data from interventional CT cases performed from March of 2016 to March of 2020. Interventional cases were filtered from diagnostic cases using study description, included descriptions were: “ablation,” “abcess drain,” “aspiration CT guided,” “biopsy CT guided,” “spine nerve root block,” and “spine sacroiliac injection.” We calculated the averages and 25th/50th/75th percentiles for: number of taps, beam energy, rotation time, and tube current. We calculated this usage data using fields obtained from our commercial dose monitoring system (DoseWatch, GE Healthcare, Chicago, IL).

### 2.5 Literature based comparison of scatter dose

We performed a literature survey on papers which quantified operator scatter dose in c‐arm based fluoroscopy and CTF. We searched using key words related to cardiology‐based catheterization lab procedures, interventional radiology procedures, and biopsy/ablation/drainage procedures in CTF. From each study, we extracted operator dose information and the values for how long the operator was exposed to x‐ray radiation. To compare dose measurements between studies using different dose metrics, we normalized all measurements to air kerma.

## 3 RESULTS

### 3.1 Experimental physician dose measurement results

**FIGURE A2:**
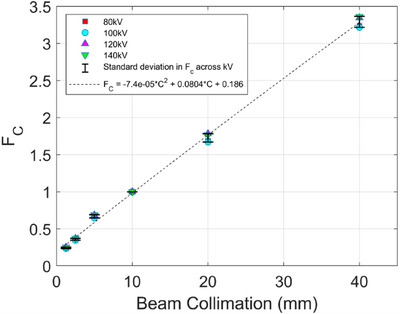
$F_{C}$ accounts for changes in operator scatter dose (i.e., entrance air kerma) with changes in beam collimation from the reference condition of 10 mm collimation. For example, the operator scatter dose at 20 mm collimation is roughly 1.5 times the dose at 10 mm collimation based on the data in Table 1. For a given measurement, the standard error is plotted but is roughly as large as the data points (i.e., the uncertainty in each measurement was smaller than the graph's symbols).

Table 1 lists physician scatter doses as a function of beam energy, collimation thickness, and inclusion of lead apron. In general, for all beam energies physician scatter dose increases with beam collimation thickness. In general, for all collimation thicknesses, physician scatter dose increases with beam energy. Appendix A includes a regression analysis of the data shown in Figure A2. Quantitative changes in physician scatter dose measured in our data with rotation time, beam energy, mA, and beam collimation thickness are shown in Table 2.

**TABLE 1:** Entrance air kerma mean in µGy at 100 mAs without and with a 0.5 mm lead apron.

Without lead apron	1.25 mm	2.5 mm	5 mm	10 mm	20 mm	40 mm
80 kV	0.467 (±0.004)	0.685 (±0.004)	1.279 (±0.005)	1.965 (±0.009)	3.315 (±0.010)	6.360 (±0.044)
100 kV	0.955 (±0.005)	1.396 (±0.011)	2.614 (±0.014)	4.030 (±0.021)	6.734 (±0.042)	12.969 (±0.071)
120 kV	1.579 (±0.013)	2.313 (±0.010)	4.302 (±0.021)	6.261 (±0.030)	11.171 (±0.046)	20.950 (±0.107)
140 kV	2.302 (±0.044)	3.365 (±0.012)	6.276 (±0.027)	9.099 (±0.039)	16.080 (±0.049)	30.607 (±0.079)
With lead apron						
80 kV	N/A*	0.004 (±0.001)	0.011 (±0.002)	0.017 (±0.002)	0.030 (±0.002)	0.059 (±0.002)
100 kV	0.014 (±0.002)	0.026 (±0.002)	0.045 (±0.003)	0.070 (±0.003)	0.122 (±0.002)	0.231 (±0.002)
120 kV	0.044 (±0.001)	0.064 (±0.003)	0.121 (±0.001)	0.186 (±0.003)	0.318 (±0.002)	0.584 (±0.004)
140 kV	0.088 (±0.001)	0.127 (±0.002)	0.233 (±0.004)	0.360 (±0.006)	0.612 (±0.012)	1.128 (±0.005)

*Note*: N/A*‐ signal was too low to produce a measurement. Standard deviation is shown in parenthesis (±SD).

**TABLE 2:** The impact of changing common iCTF acquisition parameters on physician dose is shown in this table. The percent changes in physician dose were calculated using Equation (A1) and the methodology explained in Appendix A which is based on the data presented in Table 1.

iCTF acquisition parameter	Parameter change	Percent change in physician dose	Comments
Beam energy	120 to 80 kV	−70%	*Scanner output and patient dose increase in a nonlinear fashion as beam energy increase. So does physician scatter dose*.
	120 to 100 kV	−39%	
	120 to 140 kV	45%	
Beam collimation	10 to 1.25 mm	−76%	*Physician dose does not change linearly with beam collimation because the beam collimation values are nominal values. CT vendors always irradiate wider coverages than the nominal values*.
	10 to 2.5 mm	−64%	
	10 to 5 mm	−33%	
	10 to 20 mm	73%	
	10 to 40 mm	229 %	
*Tube current*	100 to 10 mA	−90%	*Physician scatter dose is linear with tube current*.
	100 to 20 mA	−80%	
	100 to 50 mA	−50%	
	100 to 60 mA	−40%	
	100 to 200 mA	100%	
*Tube Rotation time*	1–0.3 s	−70%	*Physician scatter dose is linear with rotation time*
	1–0.5 s	−50%	

Abbreviation: iCTF, intermittent CTF.

A total of 2005 interventional CT cases were identified from which 1720 had iCTF taps series (i.e., some are done using ultrasound). The average number of iCTF taps per case was 30.1; the 25th/50th/75th percentiles in tap number were 10/24/40, respectively. The mean iCTF beam energy was 121 kV; the 25th/50th/75th percentiles in beam energy was 120/120/120 kV respectively. The average beam collimation was 6 mm; the 25th/50th/75th percentiles in beam collimation was 5/5/10 mm respectively. The average tube current was 58 mA; the 25th/50th/75th percentiles in tube current was 40/60/60 mA, respectively. The average rotation time was 0.5 s; the 25th/50th/75th percentiles in rotation time was 0.5/0.5/0.5 s. All of our iCTF taps are performed using a short scan mode in which the beam is on for 326 ms out of the 500 ms rotation time, therefore $F_{SS}=\frac{326\;ms}{500\;ms}\;$= 0.652 (discussed in Appendix A). Using “typical” acquisition techniques (all procedures) at UW‐Madison of 120 kV, 60 mA, 5 mm beam collimation, 0.5 s rotation time, lead shielded operators, short scan geometry, and iCTF tap counts of 10, 24, 30.1, and 40 (corresponding to our 25th, median, average, and 75th percentiles representing our variety in case complexity) physician dose calculated using Appendix B for 10, 24, 30.1, and 40 iCTF taps is 0.20, 0.48, 0.60, and 0.80 µGy. Unshielded statistics for UW‐Madison doses are presented in Table 1.

### 3.2 Literature survey and analysis

Table 3 lists the literature review of operator scatter doses. The mean air kerma dose rate for c‐arm based fluoroscopy was 0.23 ± 0.31 µGy/s. The mean dose rate for CTF was 0.80 ± 0.56 µGy/s. The ratio of the mean dose rate in CTF to c‐arm fluoroscopy was 3.47. The mean fluoroscopy time for c‐arm was 710.36 (±51.3) s and for CTF 22.5 (±8.3) s. The ratio of the mean c‐arm fluoroscopy to CTF time was 31.57. Overall, on an average per procedure basis, CTF yields roughly nine times lower (i.e., 31.57/3.47 = 9.1) operator scatter dose relative to c‐arm based procedures.

**TABLE 3:** iCTF usage descriptive statistics reported as “mean, 25th/50th/75th percentile.” Physician radiation dose was obtained using the procedure described in Appendix A with the acquisition parameters of the typical acquisition techniques (i.e., 50th percentile) for each procedure type and the mean number of taps. We report on a total of 2005 procedures, where only 1720 had a tap series.

Procedure type	Number procedures	Number of taps	Beam collimation (mm)	Beam energy (kV)	Tube current (mA)	Rotation time (s)	Physician dose with lead shielded (µGy)
All procedures	2005	30.1 10/24/40	6 5/5/10	121 120/120/120	58 40/60/60	0.5 0.5/0.5/0.5	0.69 ± 0.25
Ablation	100	31.9 0/0/14	8 5/10/10	122 120/120/120	72 60/60/80	0.5 0.5/0.5/0.5	1.16 ± 0.20
Abscess drain	3	3.0 0/0/7.5	10 10/10/10	120 120/120/120	100 100/100/100	0.5 0.5/0.5/0.5	0.17 ± 0.34
Aspiration CT guided	25	17.5 8.8/14/26.3	6 5/5/10	120 120/120/120	71 40/60/80	0.5 0.5/0.5/0.5	0.47 ± 0.29
Biopsy CT guided	1615	33 15/27/44	6 5/5/10	122 120/120/120	56 40/60/60	0.5 0.5/0.5/0.5	0.76 ± 0.22
Spine nerve root block	1	16 16/16/16	2 2/2/2	120 120/120/120	60 60/60/60	0.5 0.5/0.5/0.5	0.19 ± 0.0
Spine sacroiliac injection	261	13.2 6.8/12/18	5 5/5/5	117 100/120/120	69 40/60/80	0.5 0.5/0.5/0.5	0.27 ± 0.43

Abbreviation: iCTF, intermittent CTF.

## 4 DISCUSSION

Our literature review will hopefully serve to mitigate some of the apparent fear within the radiology community of CTF in‐room procedures. We anecdotally find many physicians who think c‐arm based procedures are “safer” than CTF procedures. Table 4 demonstrates the average operator dose per procedure is 9 times higher for c‐arm relative to CTF. Therefore, to obtain an average equal dose, a physician would have to perform 9 times as many CTF procedures as c‐arm procedures. These comparisons are an average, and as is visible in Table 4, all procedure types have widely varying amounts of dose and irradiation time. Physicians should always understand the dose they are receiving for the techniques and duration specific to the procedures and case complexities they perform.

**TABLE 4:** Literature review of operator dose values during fluoroscopy procedures using C‐arm and MDCT based imaging. A full version of this table with the originally reported dose values and our air kerma normalization procedure is presented in Appendix B.

Author publication year	Modality	Procedure	No. of cases	Mean fluoroscopy time (min) (±SD) or [range]	Total air kerma (µGy) without lead (±SD) or [range]	Air kerma rate without lead (µGy/s) (±SD) or [range]
Lange et al.^5^	C‐Arm single fluoroscopy	Coronary angiography	103	1.7 (±1.4)	18.7 (±23)	0.18 (±0.22)
		Percutaneous intervention	48	10.4 (±6.8)	64.3 (±67)	0.10 (±0.11)
Haga et al.^6^	C‐Arm single fluoroscopy	Coronary angiography/intervention	84	12.1 (±7.0)	35.6 (±19)	0.05 (±0.03)
Antic et al.^7^	C‐Arm single fluoroscopy	Coronary angiography	10	3.3 (±4.3)	45.5 (±48)	0.23 (±0.24)
		Coronary intervention	25	22.0 (±16.0)	90.3 (±51)	0.07 (±0.04)
Chohan et al.^8^	C‐Arm biplane fluoroscopy	Cerebral angiography	18	8.4 (±2.8)	48.3 (±123)	0.10 (±0.24)
		Cerebral interventional	6	26.8 (±6.6)	27.8 (±39)	0.02 (±0.02)
Sailer et al.^9^	C‐Arm single fluoroscopy	Infra‐renal aortic procedures	22	25.5 (±19.5)	99.4 (±124)	0.06 (±0.08)
Von Wrangel^10^	C‐Arm single fluoroscopy	Percutaneous vertebroplasty	10	16.5 [10.0–34.0]	142.1 [37–309]	0.14 [0.04–0.31]
Bahruddin et al.^11^	C‐Arm single fluoroscopy	Interventional procedures	28	2.79 [0.3–9.6]	197.4 (±138)	1.18 (±0.82)
					119.6 (±66)	0.71 (±0.39)
Ishii et al.^12^	C‐Arm Biplane fluoroscopy	Coronary angiography	18	4.3 (±1.6)	19.5 (±22)	0.08 (±0.09)
		Coronary intervention	16	21.8 (±13.3)	44.0 (±39)	0.03 (±0.03)
Stratakis et al.^13^	C‐Arm single fluoroscopy	Drainage	71	7.8 [5.7–10.95]	64	0.14
					52	0.11
Komemushi et al.^14^	C‐Arm single fluoroscopy	Percutaneous vertebroplasty	19	7.5 (±3.5)	201.6 (±136)	0.45 (±0.30)
			16	6.7 (±2.5)	73.0 (±54)	0.18 (±0.13)
Paulson et al.^15^	CT‐fluoroscopy	Biopsies	189	0.3 [0.1–0.7]	6.5 [4–30]	0.36 [0.24–1.68]
Inaba et al.^16^	CT‐fluoroscopy	Biopsies	232	0.4 (±0.3)	24.6 (±23)	1.02 (±0.95)
Inaba et al.^17^	CT‐fluoroscopy	Biopsies	95	0.3 (±0.2)	16.3	0.90
					14.9	0.83
Heusch et al.^18^	CT‐fluoroscopy	Biopsies	29	0.3 [0.1–5.6]	2.6 [0.3–145]	0.14 [0.02–8.04]
		Drainages	41	0.4 [0.1–1.1]	1.3 [0.02–35]	0.05 [0–1.45]
Kaartinen et al.^19^	CT‐fluoroscopy	Ablation	3	0.7 [0.6–1.0]	80.4 [28–107]	1.91 [0.66–2.56]
		Biopsies	57	0.3 [0.1–1.8]	22.0 [0–52]	1.22 [0–2.90]
		Drainage	16	0.3 [0.1–1.0]	23.9 [0–77]	1.33 [0–4.29]
This work (median values shown)	CT‐fluoroscopy	*All procedures*	2005	0.164 ± 0.180	24.0 ± 2.1	2.5 ± 1.2
		*Ablation*	*100*	0.173 ± 0.462	40.0 ± 3.6	3.8 ± 2.8
		*Abscess drain*	*3*	0.016 ± 0.033	6.0 ± 2.0	6.1 ± 2.4
		*Aspiration CT guided*	*25*	0.095 ± 0.075	16.8 ± 2.0	2.9 ± 0.9
		*Biopsy CT guided*	*1615*	0.179 ± 0.157	26.0 ± 1.8	2.4 ± 0.9
		*Spine nerve root block*	*1*	0.087 ± 0	6.7 ± 0.0	1.3 ± 0.0
		*Spine sacroiliac injection*	*261*	0.072 ± 0.047	10.1 ± 1.7	2.4 ± 0.8

In particular, the 9‐fold difference we found for C‐Arm and CTF procedures is based on both mean fluoroscopy times and mean dose rates. The large ratio of fluoroscopy times (31.57 C‐Arm‐to‐CTF) can be attributed to many factors including: (1) procedure complexity, (2) physician experience, (3) variations in site‐to‐site practices. Bias may be imparted if a single physician or group of physicians is selected for procedure dose comparisons. Stated differently, experience of the physician can vary at a given site where residents/fellows or physicians with decades worth of experience may perform a procedure. This can play a role given that less experienced physicians may require more time on the pedal for navigation purposes. Additionally, physician experience may also contribute to optimization of radiation reduction techniques, primarily in C‐Arm procedures where gantry orientation can vary greatly and play a large role in automatic exposure control response. However, to strictly control for this source of bias would require a well‐defined multi‐institutional evaluation of doses. Our literature review pulled manuscripts from many institutions representing many procedures and physicians. Therefore, our opinion is that this should not contribute largely to the difference in fluoroscopy time and, hence, physician dose that we report.

Each modality lends itself to different clinical indications and, hence, procedure complexity. That is, C‐Arms are well suited for device navigation through vasculature due to temporal constraints and patient motion. These procedures tend to be more complex in nature than CTF‐guided biopsies and drainages due to poorer image quality in addition to the task of device navigation through tortuous blood vessels. CTF, on the other hand, typically requires short‐distance navigation of a needle and less adjustment of device trajectory comparatively. While the dose per image frame at the detector may likely be lower for C‐Arm procedures than for CTF procedure, the complexity of navigational and therapeutic needs may contribute to longer fluoroscopy times.

Differences in fluoroscopy time between C‐Arm and CTF procedures may also be attributed to site‐to‐site variations in practice. That is, each site may have differing methods and levels of comprehensiveness for radiation safety training. The quality of training for fluoroscopy system operators can play a large role in physician dose, particularly for C‐Arm procedures, given considerations for AEC response of the imaging system. To thoroughly evaluate any systematic discrepancies in the dose comparisons pulled from literature would require a detailed comparison of technique and geometric parameters reported and stored by the system through either radiation dose structure reports (RDSRs) or controller area network (CANs).^20^ Unfortunately, this level of detail was not available in the literature we found and does pose an additional limitation of this study. Although, as state previously, the literature we pulled represents many institutions, procedures, and physicians. Therefore, we don't expect this is a significant source of error in our dose comparisons.

The methods we describe in this paper for dose calculation could be retrospectively applied to operator acquisition data to compare badge doses with our dose estimates. This is a future work for our group. Additionally, a dose monitoring vendor or scanner vendor could implement our approach to produce a real time or daily feedback of cumulative operator scatter dose. Such a display could help physicians optimize their use of CTF by allowing the physician to understand how changes to kV, beam collimation, mA, and tap number affect their dose. Based on our measurements, these important acquisition parameters have varying (and sometimes non‐linear) impacts on operator dose. With real time feedback, the operator could better find a set of acquisition parameters that provided them with interventional image utility with the smallest scatter dose. Data needed for real time or retrospective calculation is readily available in image DICOM metadata, making this approach amenable to widespread implementation if all DICOM images from an interventional CTF procedure are archived.

Inaba et al.^16^ reported an eye lens personal dose equivalent of 39.1 (±36.3) µSv which is equal to an air kerma value of 23.0 ± 21 μGy. Inaba et al.^16^ reported average scan parameters of 120 kV, 8 mm collimation, 20 mA, 0.5 s rotation time, and 26.6 s of CTF time. We can apply these parameters to Appendix A’s worksheet. The tap number for this calculation we assume equal to the fluoroscopy time divided by the rotation time, 26.6/0.5 = 53.2 taps. This assumes a single tap duration is equal to single rotation time. Inputting the Inaba et al.^16^ parameters of 20 mA, 0.5 s rotation time, 8 mm beam collimation, and 120 kV beam energy for 53.2 taps, results in a physician dose of 27.57 μGy using Appendix A. Our result calculated dose at chest height, while the Inaba et al.^16^ measurement was at the height of the eyes. Assuming an average height for a human is approximately 170 cm, an inverse square law correction to this height from the geometry assumed in our experimental set up yields an eye level dose of 21.61 µGy. This value is well within the uncertainty obtained by the author of 23.0 ± 21 μGy.

Our paper allows a physician to calculate an estimate for their own scatter dose. The dose calculation is based on our experimental data, parametrization and worksheet method described in Appendix A. In Appendix A, we also discuss how applicable our method is to other makes and models. The limits for our method's accuracy on other makes and models are within the federal regulation 21 CFR 1020.33 (c)(2)(v) guide. This federal specification on dose output accuracy states manufacturers must specify the maximum deviations. Typically, the maximum deviation is within 30% and 40%. Readers desiring to use the dose calculation worksheet presented in Appendix A should perform a comparison of their vendor's reported scatter measurements, which are provided for planning radiation protection, to the scatter measurements for the GE Discovery 750CT, which are available on GE HealthCare's Customer Documentation Portal [citation]. Example comparisons are provided in Appendix A.

A limitation of this study is our fixed location for ion chamber placement. We placed the chamber at the location an experienced interventional CTF physician at UW‐Madison usually works from. This location was modelled at chest height. For different chest heights, different body regions (e.g., the eye lens), and different distances from iso‐center the scatter dose will change. We did not quantify this change. Additionally, we only reported dose values from a single phantom size. Future works will investigate operator scatter dose from different locations and patient sizes.

Our prior work in this area measured operator scatter dose as a function of gantry position. We found that operator scatter doses increased the closer the tube was to the operator,^23^ similar to C‐arm fluoroscopy.^24^ We are aware that some CT vendors use partial angle scanning (i.e., the x‐ray beam is on for less than 360 degrees of rotation) when the operator is in a iCTF mode. The total exposure time, if reported by the vendor, can be used to scale the mAs in place of the number of taps on scanners using partial angle scanning. While the method we describe in Appendix A accounts for partial scanning, it does not account for optimal beam angles for iCTF as described in Knott et al.^23^

## 5 CONCLUSION

To our knowledge, this paper represents the first methodology for a physician using a CTF scan mode to calculate their scatter dose. The calculation uses readily available DICOM metadata and is therefore amenable to a third part dose calculation or calculation by the scanner OEM. Our literature review normalized dose rates from c‐arm based fluoroscopy and CTF demonstrated that while CTF has a roughly 3.5 times higher dose rate, an average c‐arm procedure lasts roughly 31 times longer. On average, this results in a need to perform nine CTF procedures to equal a single c‐arm based procedure operator dose.

## AUTHOR CONTRIBUTIONS

Timothy P. Szczykutowicz proposed the concepts for performing this work and oversaw its progress. Emily A. Knott performed the extensive literature reviews in this work as well as the experimental measurements. Carlos Alberto Reynoso‐Mejia contributed to theoretical baselines for deriving air kerma values from personal equivalent dose values in literature; he also performed institutional data pulls which were utilized in comparison to our literature review results. Jonathan L. Troville contributed significantly to method and result validations, including verification of the parameterization utilized in our dose algorithm. Sean D. Rose and Meghan G. Lubner contributed to the understanding and soundness of the methods and results in this work. Fred T. Lee contributed greatly to understanding of clinical impacts of this work, its technical development, and communication of results in both clinical and technical contexts. Jeffrey L. Radtke, Daniel R Anderson, and Annie Zlevor assisted with data collection and analysis. Meghan G. Lubner and J. Louis Hinshaw contributed to the clinical soundness of the results and methods communicated in the manuscript. All authors contributed to the drafting of the manuscript.

## CONFLICT OF INTEREST STATEMENT

The authors declare no conflicts of interest.

## Figures and Tables

**FIGURE A3:**
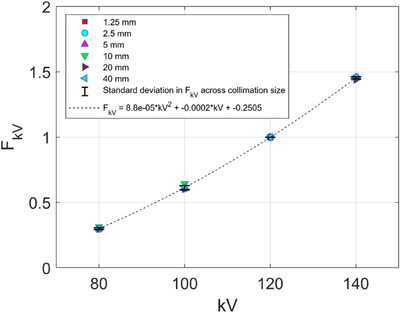
$F_{kV}$ accounts for changes in operator scatter dose (i.e., entrance air kerma) with changes in beam energy from the reference condition of 120 kV. Data is listed in Table 1. For a given measurement, the standard error is plotted but is roughly as large as the data points (i.e., the uncertainty in each measurement was smaller than the graph's symbols).

**FIGURE A4:**
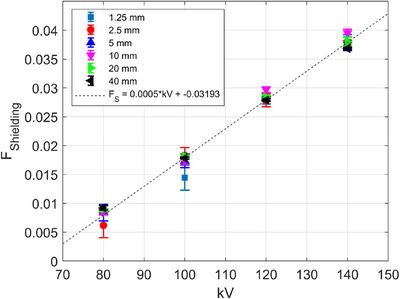
Shielding factor accounting for changes in dose (i.e., entrance air kerma) for different kV when the operator wears a lead apron with a thickness of 0.5 mm Pb. *F_S_* was obtained for each collimation size and represents the fraction of the radiation that reaches the operator penetrating through the lead apron. For example, the transmission factor of a 140 kV beam is approximately 4%, while for 80 kV the factor is 0.8%. Datapoints are shown with whiskers denoting the standard error in each measurement. The linear fit was made using the average of the six collimation sizes ratios with/without lead apron for each kV.

## APPENDIX A

### EXPERIMENTAL DETERMINATION OF THE SCATTER CORRECTION FACTORS

#### Method for operator dose calculation

We parameterized our experimental scatter results in Table 1 to allow an arbitrary CTF acquisition's operator scatter dose calculation. EAK (i.e., entrance air kerma with a backscattering medium) versus beam collimation was plotted for each kV with and without the lead apron. Plots were fit using a linear regression model. Our method scales operator scatter either higher or lower from the “reference case” of 100 mA, 1 s rotation time, 10 mm beam collimation, a full scan geometry, and 120 kV. Our parameterization accounts for changes to the number of taps, mA, rotation time, beam collimation, use of lead shielding, full versus short scan geometry, and kV. The parameterization uses the following methodology: (1) calculate the scatter correction factors (F) for each CT tap relative to the reference case, (2) multiply the scatter correction factors by the scatter dose at the reference case ($D_{ref}$), and (3) sum all the contributions from each tap.

(A1)
$$\begin{array}{ccc} & & \text{Operator Scatter Dose for n taps}\\ & & =\overset{first\;tap}{\overset{︷}{F_{mA}^{1}F_{T}^{1}F_{C}^{1}F_{kV}^{1}F_{SS}^{1}F_{S}^{1}D_{ref}}}+\overset{second\;tap}{\overset{︷}{F_{mA}^{2}F_{T}^{2}F_{C}^{2}F_{kV}^{2}F_{SS}^{2}F_{S}^{2}D_{ref}}}\\ & & +\cdots +\overset{n\;taps}{\overset{︷}{F_{mA}^{n}F_{T}^{n}F_{C}^{n}F_{kV}^{n}F_{SS}^{3}F_{S}^{3}D_{ref}}} \end{array}$$

$D_{ref}$ Is the reference operator unshielded scatter dose of 6.26 µGy, $F_{mA}^{i}$ is the mA adjustment factor relative to the reference condition, $F_{T}^{i}$ is the rotation time adjustment factor relative to the reference condition, $F_{C}^{i}$ is the collimation unshielded factor relative to the reference condition, and $F_{kV}^{i}$ is the kV adjustment factor relative to the reference condition. In non‐technical terms, Equation A1 simply scales the operator unshielded scatter dose per tap by increasing it or decreasing it relative to the “reference case” using the scatter correction factors and then adds all the contributions from each tap. The reference unshielded scatter dose condition uses 100 mA and 1 s rotation, therefore $F_{mA}$ and $F_{T}$ are defined as,

(A2)
$$F_{mA}=\frac{mA}{100mA}$$

(A3)
$$F_{T}=\frac{T}{1s}$$

$F_{T}$ accounts for changes in beam collimation width and cannot be assumed to be linear with collimation width. This is because the effects of the penumbra of the beam should have a larger influence on increasing operator scatter dose at small collimations relative to larger collimations.^3^, ^25^ Therefore, we will determine $F_{C}$ from our experimental measurements.

$F_{kV}$ accounts for changes in beam energy and cannot be assumed to be linear with beam energy. The effect of changing kV changes the total number of photons produced in a non‐linear way^26^ and when kV changes the energy distribution of photons scattering from the patient will change altering the operator scatter dose in non‐linear ways.^27^ Therefore, we will determine $F_{kV}$ from our experimental measurements.

$F_{SS}=\frac{exposure\;(ms)}{rotation\;time\;(ms)}$ accounts for changes in the total time the x‐ray beam is turned on due to the use of a short scan geometry^21^ versus a full scan geometry. Some vendors, in an effort to create higher frame rates for CTF, allow less than 360 degrees of data to be collected for use in CTF. This means the x‐ray beam is on for less than the gantry rotation time for a single iCTF tap. This factor reduces the physician exposure to account for this effect. We expect this may vary vendor to vendor and suggest interrogating DICOM headers to understand how one's specific scanner operates in CTF modes. For our GE HD 750 in “smartview” mode, the scanner uses a short scan geometry resulting in an actual exposure time of 326 ms (e.g., for this scanner model this was reported using DICOM tag “0018 1150”) for a 500 ms rotation time (e.g., for this scanner model this was reported using DICOM tag “0018 9305”). Additionally, for CTF modes that allow more than a single tap to be acquired at a time, $F_{SS}$ can take on values greater than unity. For cCTF, the exposure time is longer than the tube rotation time making multiple rotation time's worth of scan data add to physician dose but would otherwise appear as a single CTF tap. Statements in this paragraph may be vendor/model/scan mode dependent.

$F_{S}$ accounts for the reduction in physician scatter dose due to lead shielding. $F_{S}$ was calculated as the mean value of the ratios between the EAK with and without lead apron for each size of collimation. This produced 6 values of $F_{S}$ for each beam collimation at each kV, we averaged over beam collimation and then fit the resulting curve using linear regression. For those using lead aprons of a different thickness or composition than our 0.5 mm Pb aprons, the transmission factor from your apron is usually supplied by the apron manufacturer or typical transmission can be used.^22^

To experimentally determine $F_{C}$, we divided the operator scatter doses at 100 mAs for each kV by the result at 10 mm beam collimation. This produced four values of $F_{C}$ for each kV at each beam collimation, we averaged over kV and then fit the resulting curve using a second order polynomial regression. Likewise, to experimentally determine $F_{kV}$ we divided the operator scatter doses at 100 mAs for each beam collimation by the result at 120 kV. This produced 6 values of $F_{kV}$ for each beam collimation at each kV, we averaged over beam collimation and then fit the resulting curve using a second order polynomial regression.

#### Determination of $F_{C}$, $F_{kV}$, and $F_{S}$

Figures A2, A3, A4 plot EAK values normalized to the reference condition for collimation, beam energy, and beam energy respectively to obtain $F_{C}$, $F_{kV}$, and $F_{S}$. The fits from these figures define the F correction factors, with

(A4)
$$\begin{array}{ccc} F_{C} & = & -7.4*10^{-5}mm^{-2}*Collimation^{2}\\ & & +\;8.04*10^{-2}mm^{-1}*Collimation\\ & & +\;1.860*10^{-1}, \end{array}$$

(A5)
$$\begin{array}{ccc} F_{kV} & = & 8.8*10^{-5}kV^{-2}*beam\;energy^{2}\\ & & -\;2*10^{-4}kV^{-1}*beam\;energy\\ & & -\;2.505*10^{-1}, \end{array}$$

(A6)
$$F_{S}=5*10^{-4}kV^{-1}*beam\;energy-3.193*10^{-2}.$$

#### Considerations for the methods of physician dose calculation

CT scanners do use slightly different geometries and beam filtrations, which should change the operator scatter dose. Therefore, the accuracy of our method will depend on the amount of scanner‐to‐scanner variation in geometry, filtration, and beam quality. Future work will specifically address the magnitude of this variation. We are confident, however, that a single parametrization should be applicable across a wide range of CT scanners. All vendors provide room scatter diagrams that are used by medical physicists to calculate room shielding requirements. We obtained three vendor‐provided scatter diagrams from CT OEM supplied technical reference manuals. These diagrams include the scatter dose in air normalized to 100 mAs at various locations. The scatter doses in air at a point 140 cm out from isocenter at a 45‐degree angle from the CT couch were then compared for the three CT scanner models considered. From a Siemens Somatom Go.Sim at 140 kV and a 19.2 mm collimation the scatter dose in air was 5.6 µGy/100 mAs. For a GE Discovery HD 750 at 140 kV at a 40 mm collimation, the scatter dose in air was 10.4 µGy/100 mAs. For a GE LightSpeed LS 16 at 140 kV at a collimation of 20 mm, the scatter dose in air was 5.3 µGy/100 mAs. If we scale the Siemens and GE LightSpeed LS 16 by a factor of 2 to get all the results reported at roughly a 40 mm beam collimation, we see the three different models would provide 11.2, 10.4, and 10.6 µGy/100 mAs. This range of scanner model‐dependent air dose varies by less than 10%. This is well within the variations one will see in the clinic on a single scanner, as we demonstrate in the next section. Therefore, we currently believe our parametrization should be applicable across a wide range of CT scanners, albeit we would advise performing a similar check using a vendor's room scatter diagrams as we just performed here before using our parametrization to calculate operator scatter dose.

Additionally, some error is imparted through our single parameterization given that there is a co‐dependence for collimation and tube voltage. The heel effect and variation of scatter intensity with patient thickness implies that a multivariate function for collimation and tube voltage would lead to more accurate dose prediction. However, we evaluated the validity of the single‐parameterization method in this paper using a multivariate function derived from multiple regression analysis. With parameters reported in the study by Inaba et al.,^16^ mentioned in the Discussion section, we computed physician dose with our worksheet as well as the multivariate dose function. Further, we used the reported physician dose in the Inaba et al.^16^ study as a reference for dose values computed with our single‐parameterization method used in this study as well as the multivariate function. We found percent differences of 2.98% and 1.89% for the single‐parameterization and multivariate methods, respectively. Therefore, we do not expect a significant source of uncertainty from our single parameterization, and we believe our method provides a simpler and more intuitive insight into how these parameters may affect occupational dose.

We fit the scatter correction factors for collimation and beam energy using second order polynomial fits. This is an empirical decision based on our data. The realities of scatter variations present in the clinic due to operator location relative to the patient create higher uncertainties in operator dose than errors associated with making assumptions in the parameterization of our scatter correction factors. For example, our measurement probe was 85 cm from isocenter. Assuming the operator may move plus or minus 15 cm toward or away from the bore and the operator scatter dose falls off with a one over distance squared behavior, we should expect a factor of 1.47 times increase (i.e., ${\left(\frac{85cm}{70cm}\right)}^{2}=1.47$) and 0.72 times decrease (i.e., ${\left(\frac{85cm^{2}}{100cm}\right)}^{2}=0.72$) when the operator moves 15 cm toward or away from the scanner isocenter respectively. This is a clinically realistic amount of operator position uncertainty causing a change in operator scatter by more than our previously mentioned scanner model to model changes. In layman terms, the error that might be present in applying our GE HD 750 derived scatter data to a specific scanner model, is likely less than the error present in the location one physician stands relative to their colleague.

Dose worksheet for calculating operator scatter dose.

This worksheet assumes you have iCTF tap acquisitions that use identical values of tube current, rotation time, beam collimation, beam energy, scan geometry, and physician shielding. If your procedure used different combinations of the beforementioned parameters, use this worksheet for each unique set of parameters and add the resulting doses (i.e., air kerma) values.

**Step 1** Fill in the blanks labeled Rows B1‐B5
Tube current (units of milli amps (mA)) Row B1
Tube Rotation time (units of seconds) Row B2
Beam Collimation (units of mm, i.e., width of the beam at iso‐center) Row B3
Beam Energy (units of kV, i.e., tube potential) Row B4
Number of iCTF taps Row B5
**Step 2** The value obtained from inserting Row B1 into Equation (B1) Row B6
**Step 3** The value obtained from inserting Row B2 into Equation (B2) Row B7
**Step 4** The value obtained from inserting Row B3 into Equation (B3) Row B8
**Step 5** The value obtained from inserting Row B4 into Equation (B4) Row B9
**Step 6** If a lead shield is present, the value obtained from

Inserting Row B4 into Equation (B5) (see foot note 1) otherwise enter “1” Row B10

**Step 7** The value obtained from inserting Row B2 into Equation (B6) Row B11
**Step 7** The physician Entrance Air Kerma is B6xB7xB8xB9xB10*B11*6.26 µGy

Equations

(B1)
$$F_{mA}=\frac{Value}{100mA}$$

(B2)
$$F_{T}=\frac{Value}{1s}\cdot$$

(B3)
$$\begin{array}{ccc} F_{c} & = & -7.4*10^{-5}mm^{-2}*Value^{2}\\ & & +\;8.04*10^{-2}mm^{-1}*Value\\ & & +\;1.860*10^{-1} \end{array}$$

(B4)
$$\begin{array}{ccc} F_{kV} & = & 8.8*10^{-5}kV^{-2}*Value^{2}\\ & & -\;2*10^{-4}kV^{-1}*Value\\ & & -\;2.505*10^{-1},\mathrm{and} \end{array}$$

(B5)
$$Fs=5*10^{-4}kV^{-1}*Value-3.193*10^{-2}\cdot$$

(B6)
$$F_{ss}=\frac{\exp osure\;time\;(s)}{Value}\;\left(see\;foot\;note\;2\right)$$

**Footnote 1** Assumes a lead apron thickness of 0.5 mm Pb equivalent. If your apron uses another Pb thickness or material, insert your apron's transmission factor.

**Footnote 2** The actual beam on time for a single tap divided by the rotation time. This accounts for short scan iCTF acquisitions where the beam on time is less than the rotation time or “lead foot” physicians using cCTF modes where the beam is on for multiple tube rotations.

## APPENDIX B

### NORMALIZATION OF LITERATURE REVIEW DOSE METRICS

We found dose values reported using units of personal dose equivalents Hp(0.07) [µSv], Hp (3) [µSv], Hp (10) [µSv], and surface air kerma (including backscatter) [µGy]. To convert the operational quantities Hp(10), Hp(3), and Hp(0.07) to air kerma, we used: (a) a radiation weighting factor of 1, (b) the average energy values of the energy spectra from a c‐arm reported by Nowak et al.^28^ and MDCT reported by Berg,^29^ and (c) the analytical function relating Hp to surface air kerma for c‐arm procedures published by Kanti et al.^30^ (Hp/Ka = 1.62, 1.52, and 1.71 for Hp(0.07), Hp(3) and Hp(10) respectively for thorax level, and Hp/Ka = 1.67, 1.56, and 1.80 respectively for eyes level) and for MDCT published by Kanti et al.^30^ and Berg^29^ (Hp/Ka = 1.55, 1.46, and 1.59 for Hp(0.07), Hp(3), and Hp(10) respectively for 120 kV and eye level). The surface air Kerma rate was obtained by dividing the total surface air kerma by the mean fluoroscopy time. Table B1 lists the results of the literature review of operator dose values during fluoroscopy procedures using C‐arm and MDCT based imaging. This table comprehensively lists a wide variety of procedure types using both C‐Arm and interventional CT fluoroscopy. Furthermore, the table highlights average fluoroscopy times, dose metrics (personal dose equivalents: Hp(0.01), Hp(3), Hp(10), or surface air kerma including backscatter), as well as derived physician surface air kerma/air kerma rate without lead shielding.

**TABLE B1:** Literature review of operator dose values during fluoroscopy procedures using C‐arm and MDCT based imaging. Dose units are as follows: ^a^ Hp(0.07) [µSv], ^b^ Hp (3) [µSv], ^c^ Hp (10) [µSv], and ^k^ air kerma [µGy], and were not modified from their original source references. ^Ɨ^ Data was initially reported as a cumulative dose of several months, we divided the reported cumulative dose by the number of cases.

Author publication year	modality	Procedure	No of cases	Mean fluoroscopy time (min) (±SD) or [range]	Dose values without lead per procedure (quantity and unit change per study, see table notes) (±SD) or [range]	Total Air Kerma (µGy) without lead (±SD) or [range]	Air Kerma rate without lead (µGy/s) (±SD) or [range]
Lange et al.^5^	C‐Arm single fluoroscopy	Coronary angiography	103	1.7 (±1.4)	32 (±39) ^c^	18.7 (±23)	0.18 (±0.22)
		Percutaneous intervention	48	10.4 (±6.8)	110 (±115) ^c^	64.3 (±67)	0.10 (±0.11)
Haaga et al.^6^	C‐Arm single fluoroscopy	Coronary angiography/ intervention	84	12.1 (±7.0)	59.5 (±31) ^a,Ɨ^	35.6 (±19)	0.05 (±0.03)
Antic et al.^7^	C‐Arm single fluoroscopy	Coronary angiography	10	3.3 (±4.3)	71 (±75) ^b^	45.5 (±48)	0.23 (±0.24)
		Coronary intervention	25	22.0 (±16.0)	141 (±80) ^b^	90.3 (±51)	0.07 (±0.04)
Chohan et al.^8^	C‐Arm biplane fluoroscopy	cerebral angiography	18	8.4 (±2.8)	87 (±222) ^c^	48.3 (±123)	0.10 (±0.24)
		cerebral interventional	6	26.8 (±6.6)	50 (±70) ^c^	27.8 (±39)	0.02 (±0.02)
Sailer et al.^9^	C‐Arm single fluoroscopy	infra‐renal aortic procedures	22	25.5 (±19.5)	170 (±210) ^c^	99.4 (±124)	0.06 (±0.08)
Von Wrangel^10^	C‐Arm single fluoroscopy	Percutaneous Vertebroplasty	10	16.5 [10.0–34.0]	230 [60–500] ^a^	142.1 [37–309]	0.14 [0.04‐0.31]
Bahruddin et al.^11^	C‐Arm single fluoroscopy	interventional procedures	28	2.79 [0.3–9.6]	330 (±230) ^a^	197.4 (±138)	1.18 (±0.82)
					200 (±110) ^a^	119.6 (±66)	0.71 (±0.39)
Ishii et al.^12^	C‐Arm Biplane fluoroscopy	coronary angiography	18	4.3 (±1.6)	35.2 (±40.1) ^c^	19.5 (±22)	0.08 (±0.09)
		coronary intervention	16	21.8 (±13.3)	79.3 (±69.9) ^c^	44.0 (±39)	0.03 (±0.03)
Stratakis et al.^13^	C‐Arm single fluoroscopy	Drainage	71	7.8 [5.7‐10.95]	64 ^k^	64	0.14
					52 ^k^	52	0.11
Komemushi et al. (2005)^14^	C‐Arm single fluoroscopy	Percutaneous vertebroplasty	19	7.5 (±3.5)	320.8 (±232.2) ^c^	201.6 (±136)	0.45 (±0.30)
			16	6.7 (±2.5)	116.2 (±92.8) ^c^	73.0 (±54)	0.18 (±0.13)
Paulson et al.^15^	CT‐fluoroscopy	biopsies	189	0.3 [0.1‐0.7]	10.3 [7–48] ^c^	6.5 [4–30]	0.36 [0.24‐1.68]
Inaba et al.^16^	CT‐fluoroscopy	biopsies	232	0.4 (±0.3)	39.1 (±36.3) ^c^	24.6 (±23)	1.02 (±0.95)
Inaba et al.^17^	CT‐fluoroscopy	biopsies	95	0.3 (±0.2)	23.7 ^b Ɨ^	16.3	0.90
					21.7 ^b Ɨ^	14.9	0.83
Heusch et al.^18^	CT‐fluoroscopy	biopsies	29	0.3 [0.1–5.6]	3.9 [0.5−218.9] ^a^	2.6 [0.3–145]	0.14 [0.02–8.04]
		Drainages	41	0.4 [0.1–1.1]	1.9 [0.03−52.6] ^a^	1.3 [0.02–35]	0.05 [0–1.45]
Kaartinen et al.^19^	CT‐fluoroscopy	Ablation	3	0.7 [0.6–1.0]	128 [44−171] ^c^	80.4 [28–107]	1.91 [0.66–2.56]
		Biopsies	57	0.3 [0.1–1.8]	35 [0−83] ^c^	22.0 [0–52]	1.22 [0–2.90]
		Drainage	16	0.3 [0.1–1.0]	38 [0–123] ^c^	23.9 [0–77]	1.33 [0–4.29]
This work (median values shown)	CT‐fluoroscopy	*All procedures*	2005	0.164 (±0.180)	24.0 ± 2.1^k^	24.0 ± 2.1	2.5 ± 1.2
		*Ablation*	*100*	0.173 (±0.462)	40.0 ± 3.6 ^k^	40.0 ± 3.6	3.8 ± 2.8
		*Abscess drain*	*3*	0.016 (±0.033)	6.0 ± 2.0 ^k^	6.0 ± 2.0	6.1 ± 2.4
		*Aspiration CT guided*	*25*	0.095 (±0.075)	16.8 ± 2.0 ^k^	16.8 ± 2.0	2.9 ± 0.9
		*Biopsy CT guided*	*1615*	0.179 (±0.157)	26.0 ± 1.8 ^k^	26.0 ± 1.8	2.4 ± 0.9
		*Spine nerve root block*	*1*	0.087 (±0.000)	6.7 ± 0.0 ^k^	6.7 ± 0.0	1.3 ± 0.0
		*Spine sacroiliac injection*	*261*	0.072 (±0.047)	10.1 ± 1.7 ^k^	10.1 ± 1.7	2.4 ± 0.8

## References

[1] Haaga JR , Alfidi RJ . Precise biopsy localization by computed tomography. Radiology. 1976;118(3):603‐607. 1251009 10.1148/118.3.603

[2] Kalender WA , Seissler W , Klotz E , Vock P . Spiral volumetric CT with single‐breath‐hold technique, continuous transport, and continuous scanner rotation. Radiology. 1990;176(1):181‐183. 2353088 10.1148/radiology.176.1.2353088

[3] Szczykutowicz T . CT Handbook: Optimizing Protocols for Today's Feature‐Rich Scanners. Medical Physics Publishing; 2020.

[4] Stoeckelhuber BM , Schulz E , Melchert UH . Procedures, spectrum and radiation exposure in CT‐fluoroscopy. Rontgenpraxis; Zeitschrift fur Radiologische Technik. 2003;55(2):51‐57. 14618963

[5] Lange HW , von Boetticher H . Randomized comparison of operator radiation exposure during coronary angiography and intervention by radial or femoral approach. Catheter Cardiovasc Interv. 2006;67(1):12‐16. 10.1002/ccd.20451 16331696

[6] Haga Y , Chida K , Kaga Y , Sota M , Meguro T , Zuguchi M . Occupational eye dose in interventional cardiology procedures. Sci Rep. 2017;7(1):569. 10.1038/s41598-017-00556-3 28373715 PMC5428664

[7] Antic V , Ciraj‐Bjelac O , Rehani M , Aleksandric S , Arandjic D , Ostojic M . Eye lens dosimetry in interventional cardiology: results of staff dose measurements and link to patient dose levels. Radiat Prot Dosim. 2013;154(3):276‐284. 10.1093/rpd/ncs236 23152146

[8] Chohan MO , Sandoval D , Buchan A , Murray‐Krezan C , Taylor CL . Cranial radiation exposure during cerebral catheter angiography. J Neurointerv Surg. 2014;6(8):633‐636. 10.1136/neurintsurg-2013-010909 24062257

[9] Sailer AM , Schurink GW , Bol ME , et al. Occupational radiation exposure during endovascular aortic repair. Cardiovasc Intervent Radiol. 2015;38(4):827‐832. 10.1007/s00270-014-1025-8 25476871

[10] von Wrangel A , Cederblad Å , Rodriguez‐Catarino M . Fluoroscopically guided percutaneous vertebroplasty: assessment of radiation doses and implementation of procedural routines to reduce operator exposure. Acta Radiol. 2009;50(5):490‐496. 10.1080/02841850902855391 19363715

[11] Bahruddin NA , Hashim S , Karim MKA , et al. Radiation dose to physicians’ eye lens during interventional radiology. J Phys Conf Ser. 2016;694:012035. 10.1088/1742-6596/694/1/012035

[12] Ishii H , hida K , Satsurai K , et al. Occupational eye dose correlation with neck dose and patient‐related quantities in interventional cardiology procedures. Radiol Phys Technol. 2022;15(1):54‐62. 10.1007/s12194-022-00650-w 35067903

[13] Stratakis J , Damilakis J , Hatzidakis A , Theocharopoulos N , Gourtsoyiannis N . Occupational radiation exposure from fluoroscopically guided percutaneous transhepatic biliary procedures. J Vasc Interv Radiol. 2006;17(5):863‐871. 10.1097/01.RVI.0000217959.86251.11 16687753

[14] Komemushi A , Tanigawa N , Kariya S , Kojima H , Shomura Y , Sawada S . Radiation exposure to operators during vertebroplasty. J Vasc Interv Radiol. 2005;6(10):1327‐1332. 10.1097/01.RVI.0000179794.65662.01 16221903

[15] Paulson EK , Sheafor DH , Enterline DS , McAdams HP , Yoshizumi TT . CT fluoroscopy‐guided interventional procedures: techniques and radiation dose to radiologists. Radiology. 2001;220(1):161‐167. 10.1148/radiology.220.1.r01jl29161 11425990

[16] Inaba Y , Hitachi S , Watanuki M , Chida K . Occupational radiation dose to eye lenses in CT‐guided interventions using MDCT‐fluoroscopy. Diagnostics (Basel). 2021;11(4):646. 10.3390/diagnostics11040646 33918341 PMC8065869

[17] Inaba Y , Hitachi S , Watanuki M , Chida K . Radiation eye dose for physicians in CT fluoroscopy‐guided biopsy. Tomography. 2022;8(1):438‐446. 10.3390/tomography8010036 35202201 PMC8878526

[18] Heusch P , Kröpil P , Buchbender C , et al. Radiation exposure of the radiologist's eye lens during CT‐guided interventions. Acta Radiol. 2014;55(1):86‐90. 10.1177/0284185113493222 23884839

[19] Kaartinen S , Husso M , Matikka H . Operator's eye lens dose in computed tomography‐guided interventions. Eur Radiol. 2021;31(6):4377‐4385. 10.1007/s00330-020-07576-0/Published 33349894 PMC8128838

[20] Troville J , Rudin S , Bednarek DR . A prototype software system for intra‐procedural staff dose monitoring and virtual reality training for fluoroscopically guided interventional procedures. J Digit Imaging. 2023;36(3):1091‐1109. 36828961 10.1007/s10278-023-00790-4 PMC10287622

[21] Parker DL . Optimal short scan convolution reconstruction for fan beam CT. Med Phys. 1982;9(2):254‐257. 7087912 10.1118/1.595078

[22] Franken Y , Guidance on the use of protective lead aprons in medical radiology protection efficiency and correction factors for personal dosimetry. In 6th European ALARA Network Workshop. Occupational Exposure Optimisation in the Medical Field and Radiopharmaceutical Industry. Madrid, 2002. October 23–25, 2002.

[23] Knott EA , Rose SD , Wagner MG , et al. CT fluoroscopy for image‐guided procedures: physician radiation dose during full‐rotation and partial‐angle CT scanning. J Vasc Interv Radiol. 2021;32(3):439‐446. 10.1016/j.jvir.2020.10.033 33414069

[24] Schueler BA , Vrieze TJ , Bjarnason H , Stanson AW . An investigation of operator exposure in interventional radiology. Radiographics. 2006;26(5):1533‐1541. 16973780 10.1148/rg.265055127

[25] Jackson SR , Ahmad S , Hu Y , Ruan C . Evaluation of different techniques for CT radiation profile width measurement. J Appl Clin Med Phys. 2013;14(4):227‐237. 10.1120/jacmp.v14i4.4122 PMC5714537 23835377

[26] Boone JM , Seibert JA . An accurate method for computer‐generating tungsten anode x‐ray spectra from 30 to 140 kV. Med Phys. 1997;24(11):1661‐1670. 9394272 10.1118/1.597953

[27] Attix FH . Introduction to Radiological Physics and Radiation Dosimetry. Wiley; 1986.

[28] Nowak M , Carbonez P , Krauss M , Verdun FR , Damet J . Characterisation and mapping of scattered radiation fields in interventional radiology theatres. Sci Rep. 2020;10(1):18754. 10.1038/s41598-020-75257-5 33127938 PMC7599331

[29] Berg H . Radiation Exposure to Personnel during CT Procedures. 2018. (Thesis). Retrieved from https://urn.kb.se/resolve?urn=urn:nbn:se:kau:diva‐68152

[30] Al Kanti H , El Hajjaji O , El Bardouni T . Conversion coefficients from fluence and air kerma to personal dose equivalent for monoenergetic photons using analytical fit and Monte Carlo simulation. Pol J Med Phys Eng. 2020;26(1):31‐44. 10.2478/pjmpe-2020-0004

